# Magnetofection of miR-21 promoted by electromagnetic field and iron oxide nanoparticles via the p38 MAPK pathway contributes to osteogenesis and angiogenesis for intervertebral fusion

**DOI:** 10.1186/s12951-023-01789-3

**Published:** 2023-01-25

**Authors:** Tianqi Wang, Hongqi Zhao, Shaoze Jing, Yang Fan, Gaohong Sheng, Qing Ding, Chaoxu Liu, Hua Wu, Yang Liu

**Affiliations:** 1 Department of Orthopedics, Tongji Hospital, Tongji Medical College, Huazhong University of Science and Technology, Wuhan, 430030 China; 2 Third Hospital of Shanxi Medical University, Shanxi Bethune Hospital, Shanxi Academy of Medical Sciences, Tongji Shanxi Hospital, Taiyuan, 030032 China; 3 Department of Geriatrics, Tongji Hospital, Tongji Medical College, Huazhong University of Science and Technology, Wuhan, 430030 China; 4 Department of Pediatrics, Tongji Hospital, Tongji Medical College, Huazhong University of Science and Technology, Wuhan, 430030 China

**Keywords:** Bone tissue engineering, Electromagnetic field, Gene therapy, Iron oxide nanoparticles, Magnetofection

## Abstract

**Background:**

Magnetofection-mediated gene delivery shows great therapeutic potential through the regulation of the direction and degree of differentiation. Lumbar degenerative disc disease (DDD) is a serious global orthopaedic problem. However, even though intervertebral fusion is the gold standard for the treatment of DDD, its therapeutic effect is unsatisfactory. Here, we described a novel magnetofection system for delivering therapeutic miRNAs to promote osteogenesis and angiogenesis in patients with lumbar DDD.

**Results:**

Co-stimulation with electromagnetic field (EMF) and iron oxide nanoparticles (IONPs) enhanced magnetofection efficiency significantly. Moreover, in vitro, magnetofection of miR-21 into bone marrow mesenchymal stem cells (BMSCs) and human umbilical endothelial cells (HUVECs) influenced their cellular behaviour and promoted osteogenesis and angiogenesis. Then, gene-edited seed cells were planted onto polycaprolactone (PCL) and hydroxyapatite (HA) scaffolds (PCL/HA scaffolds) and evolved into the ideal tissue-engineered bone to promote intervertebral fusion. Finally, our results showed that EMF and polyethyleneimine (PEI)@IONPs were enhancing transfection efficiency by activating the p38 MAPK pathway.

**Conclusion:**

Our findings illustrate that a magnetofection system for delivering miR-21 into BMSCs and HUVECs promoted osteogenesis and angiogenesis in vitro and in vivo and that magnetofection transfection efficiency improved significantly under the co-stimulation of EMF and IONPs. Moreover, it relied on the activation of p38 MAPK pathway. This magnetofection system could be a promising therapeutic approach for various orthopaedic diseases.

**Graphical Abstract:**

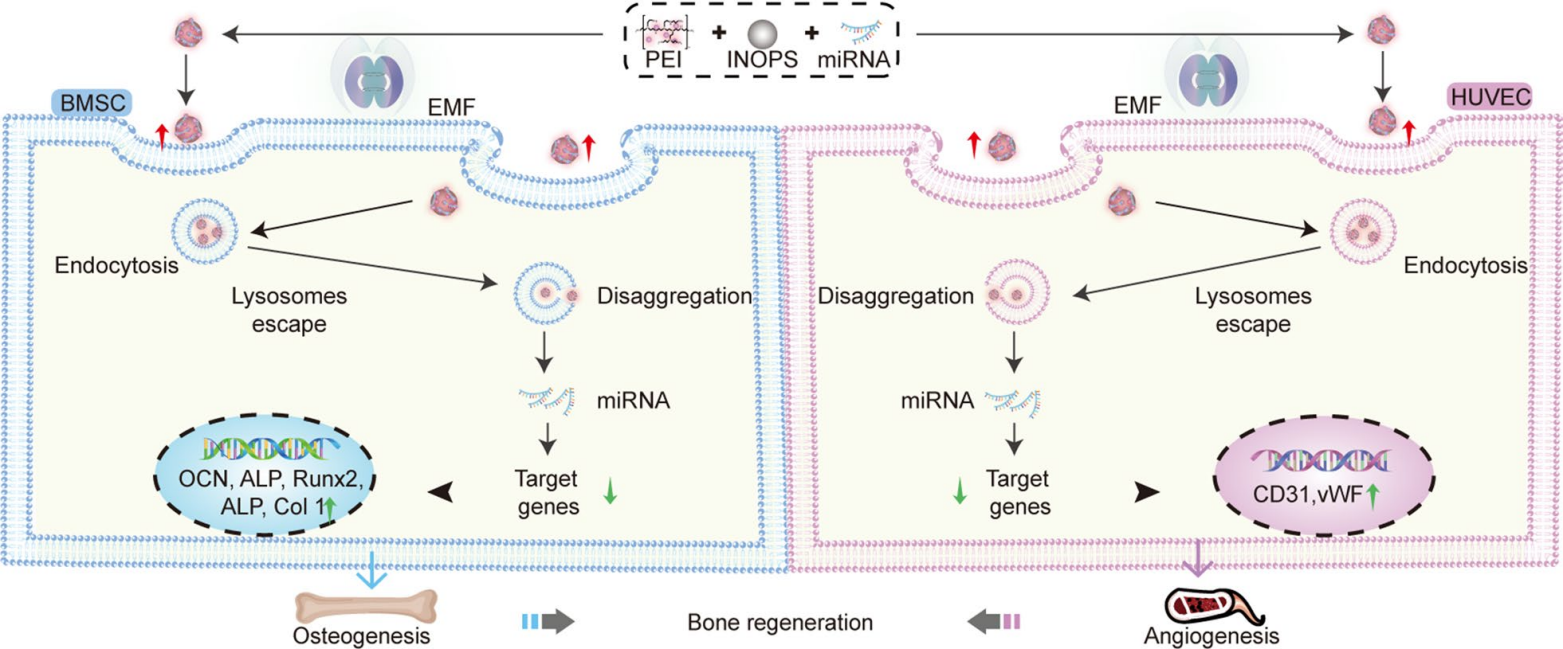

## Background

Lumbar degenerative disc disease (DDD) is a common clinical condition that causes low back pain from damaged discs in the spine [1, 2]. DDD is one of the most common reasons people seek medical assistance, causing substantial health, social, and economic burdens [3, 4]. Currently, posterior or transforaminal lumbar interbody fusion is the gold-standard therapy for DDD. It involves creating a stabilized, decompressed interbody fusion structure to relieve back pain [5–8]. Grafts (mainly autografts) are planted between two adjacent interbodies to promote lumbar interbody fusion. However, the inefficiency and low success rate of this method, together with limited autograft supply and various complications, including immunosuppression and infection, restrain its further clinical practice. Bone tissue engineering (BTE) methods have emerged as a next-generation strategy to address the abovementioned drawbacks. In general, seed cells, growth factors, and scaffolds are the three fundamental elements of BTE.

Gene therapy is a technique that focuses on the genetic modification of cells, and it can modulate the direction and degree of seed cell differentiation [9–13]. To up- or down-regulate the expression of specific proteins, exogenous genetic materials (such as DNA, siRNA, and microRNA) need to be transported to target cells through viral or nonviral vectors [14, 15]. Despite their high efficiency, viral vectors come with multiple complications, such as their immunostimulatory potential and toxicity [16–18]. As a result, nonviral vectors (such as peptides [19], iron oxide nanoparticles (IONPs) [20, 21], and polymers [22]) and physical transfection methods (such as electroporation, ultrasound, and magnetic field) have been developed and applied due to their advantages, including low immunogenicity, easy production, and low costs.

Among these, transfecting IONP-bound genetic materials into target seed cells driven by magnetic field has been commercialized and termed as magnetofection [23–25]. Magnetofection consists of two essential elements: a magnetic field, as in the static magnetic field generated by magnets, and IONPs. Electromagnetic fields (EMF), combinations of electric and magnetic fields, have achieved a great deal of attention owing to their non-invasive features [26]. Polyethyleneimine (PEI) is a conventional and well-established transfection agent [25, 27]. In this study, we performed a comprehensive strategy that substituted an EMF for a static magnetic field and also coated PEI on the IONPs to enhance the efficiency of magnetofection.

MicroRNAs (miRNAs) act as post-transcriptional regulators [28, 29] and emerge as important osteogenesis and angiogenesis regulators by identifying and targeting specific pathogenic genes, which could be potential therapeutic candidates [30, 31]. Many miRNAs are reported to enhance osteogenic differentiation, such as miR-26a [32], miR-29b [33] and miR-21b [34, 35]. Therefore, the employment of miRNA in magnetofection methods seems like a feasible and promising strategy for bone regeneration.

Scaffolds are another key aspect of BTE [36]. Immune response, cellular metabolisms, and extracellular environment can become obstacles to further applications of direct cell therapy [37, 38]. Nonetheless, biocompatible scaffolds play a prominent role in BTE by protecting and delivering seed cells effectively [39, 40]. As reported, polycaprolactone (PCL) and hydroxyapatite (HA) scaffolds have been produced and widely applied in BTE due to their good biodegradability and excellent mechanical performance [41–43].

Many studies reported that the p38 MAPK signalling pathway was strongly associated with cytoskeletal rearrangement and endocytosis. Inhibition of P38 MAPK by SB239063 has been proven to reduce the cellular uptake of IONPs [44], while activation of the p38 MAPK signalling pathway is related to enhanced endocytosis [45]. Upon activation by p38, MAPK, one of serine/threonine protein kinases family, can regulate the activity of Hsp27 to mediate the cytoskeleton rearrangement [46, 47]. In our previous studies, EMF exposure activated P38 MAPK by promoting p38 phosphorylation [48, 49].

## Results

### Characterization of PEI@IONPs and magnetic conditions

**Fig. 1:**
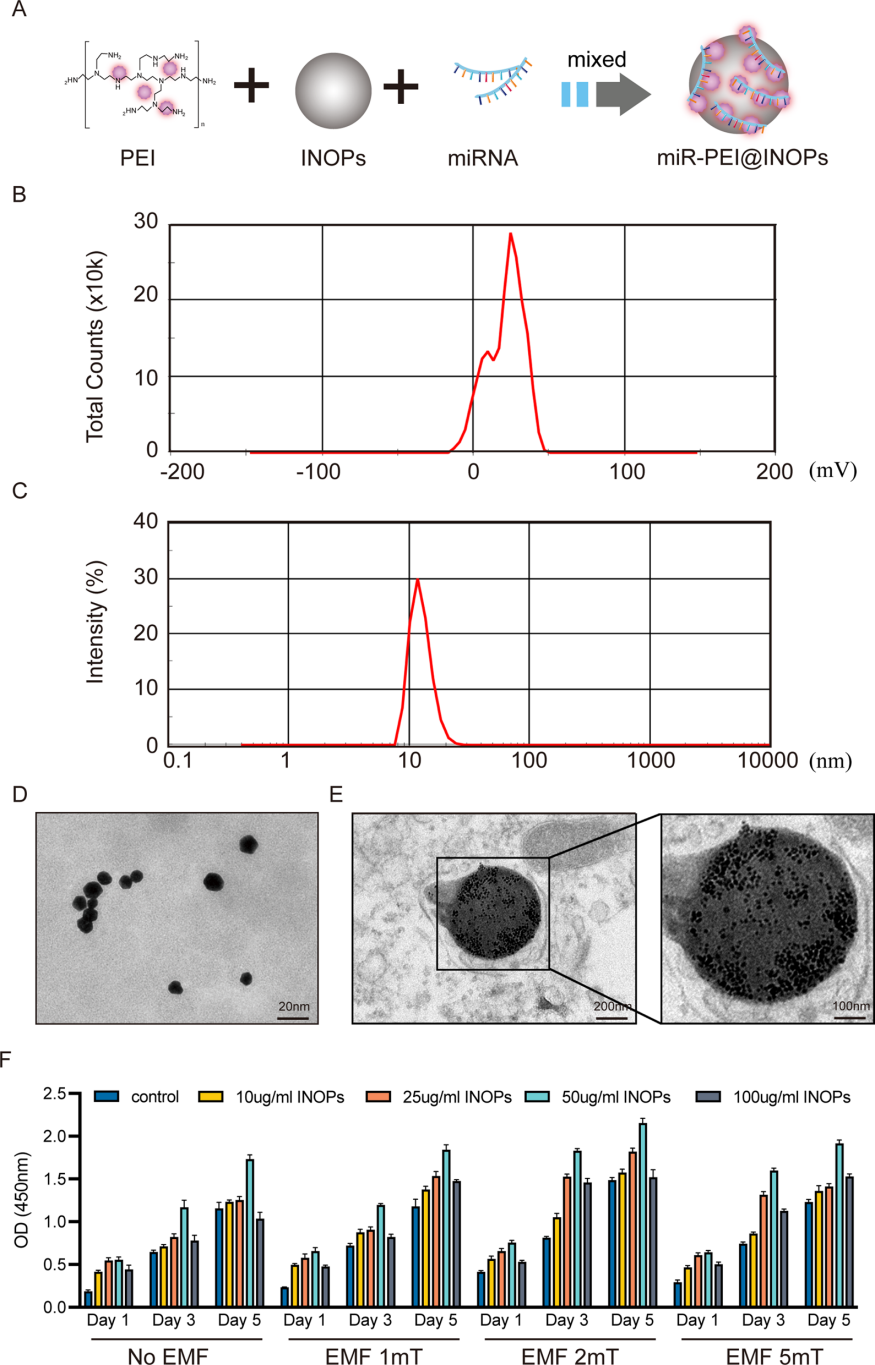
Design and characterization of PEI@IONPs and corresponding magnetofection complexes systems.** A** Schematic illustration of synthesis process of magnetofection complexes systems (miR-PEI@IONPs) using polyethyleneimine (PEI)-coated iron oxide nanoparticles (IONPs) to deliver miRNAs. **B–D** Zeta potential (**B**), diameter (**C**) and morphology (**D**) of the PEI@IONPs. **E** Transmitting electron microscope images indicating the internalisation of IONPs by seed cells. The image on the right is a magnification of the marked box on the left. **F** Cell counting kit-8 results of seed cells treated with various conditions (IONPs concentration: 0, 10, 25, 50, 100 µg/mL and EMF intensity: 0, 1, 2 or 5 mT)

IONPs and magnetic conditions are the fundamental components of magnetofection (Fig. 1A). PEI was chosen to coat IONPs owing to its excellent intracellular gene delivery and to its proton sponge capability, which facilitates release from endosomes. After PEI modification, PEI@IONPs showed a positive zeta potential of 30.2 mV (Fig. 1B) and an average particle size of 10.31 nm (Fig. 1C). Transmitting electron microscope (TEM) images showed that PEI@IONPs had a sphere-like morphology (Fig. 1D). After internalisation of seed cells, PEI@IONPs were uniformly distributed across the cytoplasm and did not influence the cellular morphology or function (Fig. 1E). To detect the cytotoxicity with different PEI@IONPs concentrations and different EMF intensities, seed cells were cultured in a medium treated with 0, 10, 25, 50, or 100 µg/mL PEI@IONPs with an exposure of 0, 1, 2, or 5 mT EMF. The cell counting kit-8 (CCK-8) results indicated that the combination of 50 µg/mL PEI@IONPs and 2 mT EMF provided the best fit for the growth and proliferation of seed cells (Fig. 1F).

### Optimal magnetofection conditions for the miR-PEI@IONPs

**Fig. 2:**
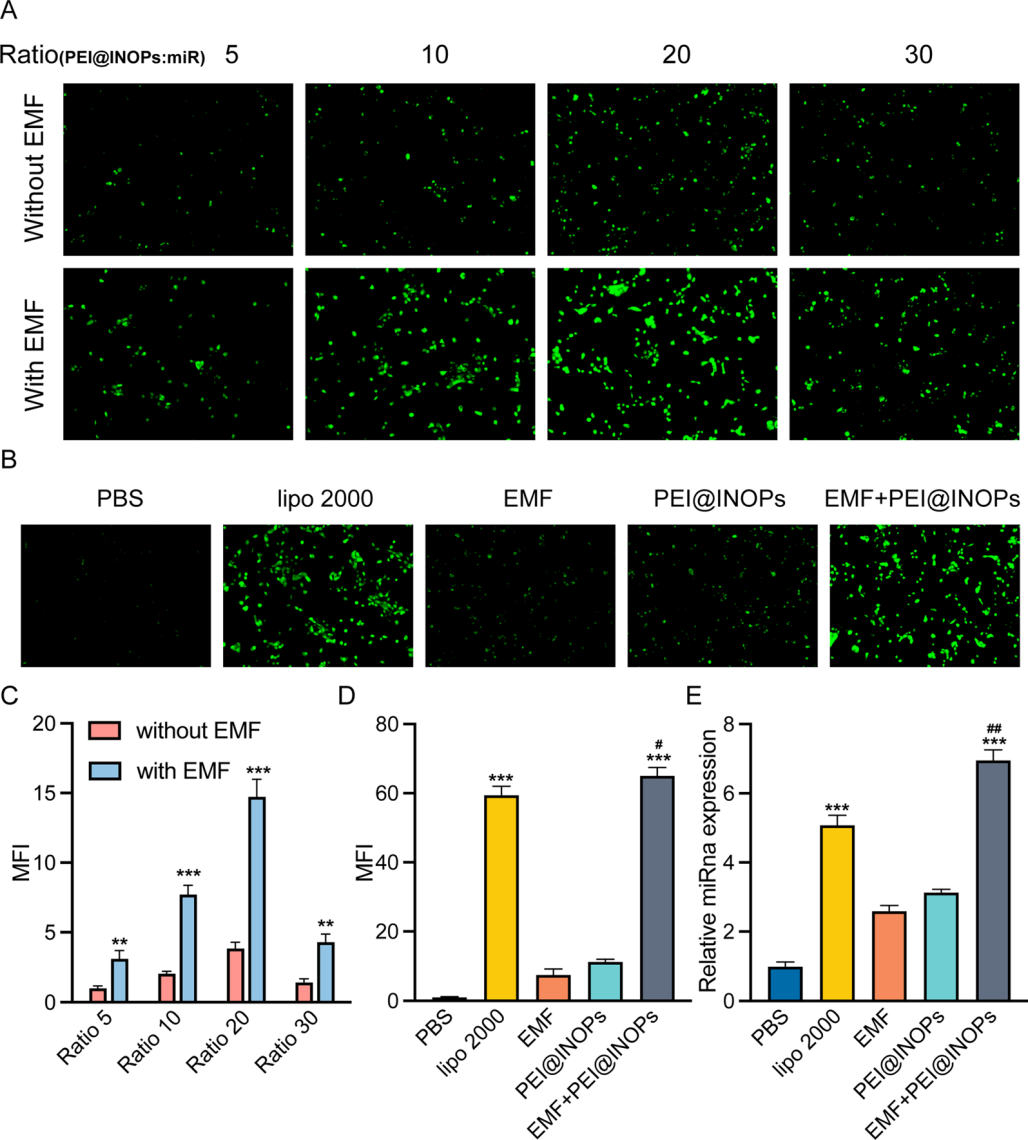
Different transfection efficiencies under various magnetofection conditions.** A** Fluorescence microscope images showing different transfection efficiencies according to different distant ratios of PEI@IONPs:miRNAs [5, 10, 20, or 30, with or without electromagnetic field (EMF) stimulation]. **C** Semi-quantifications of mean fluorescence intensity under those various conditions. **B**,** D** Transfection efficiency under various treatments [PBS, lipofectamine 2000 (lipo 2000), EMF, PEI@INPs, EMF + PEI@IONPs] and corresponding mean fluorescence intensity. **E** Relative miRNA expression levels of miR-21 under various treatments (PBS, lipo 2000, EMF, PEI@INPs, EMF + PEI@IONPs). *p < 0.05, **p < 0.01, ***p < 0.001 compared to the PBS group; ^#^p < 0.05, ^##^p < 0.01 compared to the lipo 2000 group

Transfection efficiency under different conditions (distant ratio of PEI@IONPs:miRNA and various treatments) was assessed by the mean fluorescence intensity (MFI) of GFP in the 293T cell line. It was found that EMF significantly increased transfection efficiency, especially with a ratio PEI@INOPs: miRna at 20 (Fig. 2A). The EMF effect was evident as its MFI promoted almost 2-fold compared to that of only PEI@IONPs (Fig. 2C). Therefore, we selected a PEI@IONPs:miRNA ratio of 20 for further experiments. Furthermore, the EMF-PEI@IONPs group increased transfection efficiency by approximately 10% compared to that of the lipofectamine 2000 (lipo 2000) group (Fig. 2B and D). To observe whether the magnetofection of miRNA was successful, we employed qPCR to detect miRNA expression levels. The results showed that the EMF-PEI@IONPs group had a higher miR-21 expression level than that of the other groups (PBS, lipo 2000, EMF, and PEI@IONPs groups) (Fig. 2E). In conclusion, the magnetofection complexes performed the best transfection efficiency at the ratio of 20 and EMF could significantly enhance transfection efficiency.

### Magnetofection of miR-21 enhances osteogenesis in vitro

**Fig. 3:**
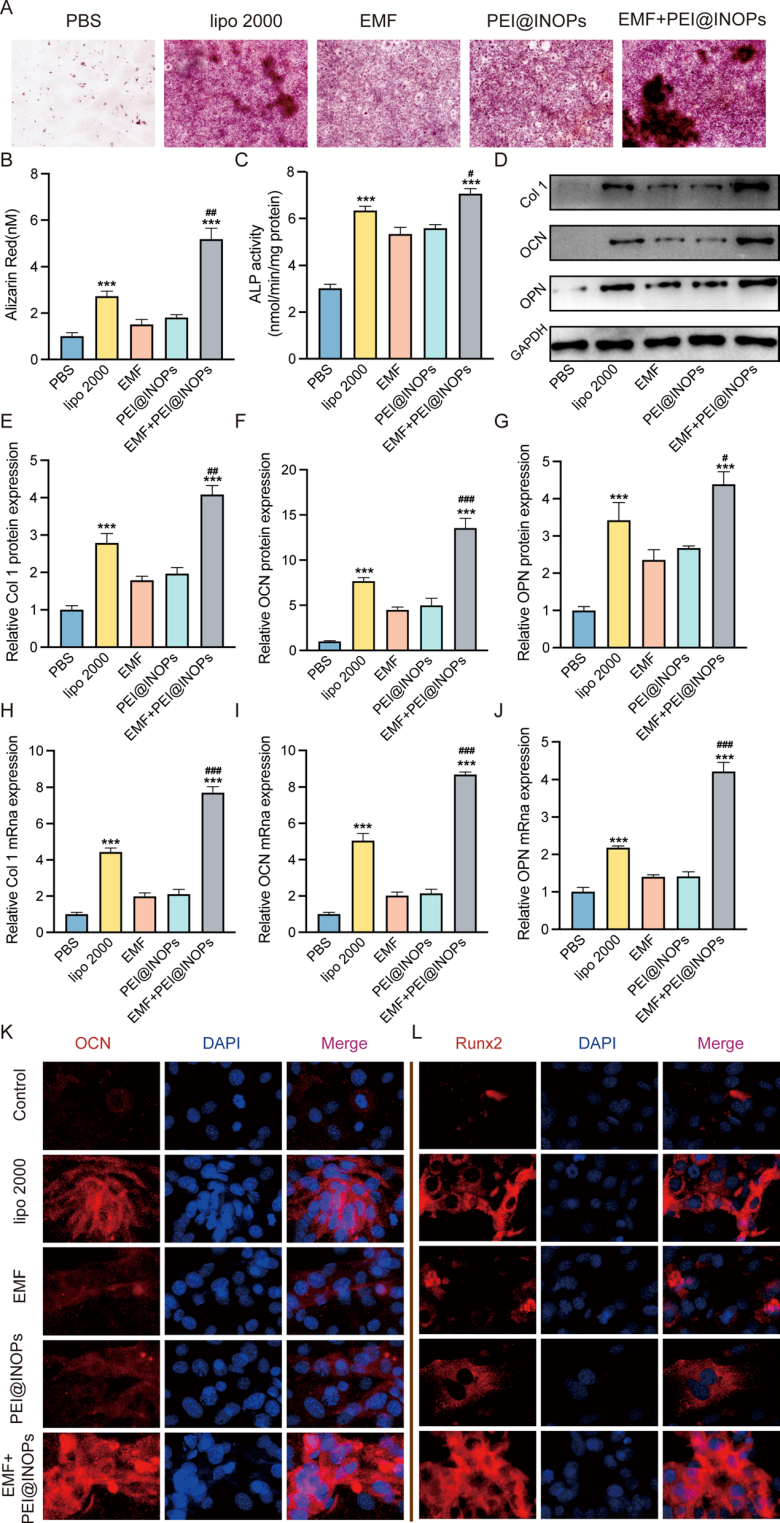
Magnetofection of miR-21 promoted osteogenesis.** A**,** B** Alizarin red S (ARS) staining pictures 14 days after different treatments and the corresponding semi-quantification analysis. **C** Quantification analysis of alkaline phosphatase (ALP) activity 7 days after treatment. **D–G** Western Blotting analysis of Col 1, OCN, and OPN as well as corresponding semi-quantifications of protein expression levels of Col 1 (**E**), OCN (**F**) and OPN (**G**). **H–J** Relative mRNA expression levels of Col 1 (**H**), OCN (**I**), and OPN (**J**). **K**,** L** Immunofluorescence images of OCN (**K**) and Runx2 (**L**) after various treatments (PBS, lipofectamine 2000 (lipo 2000), electromagnetic field (EMF), PEI@INPs, EMF + PEI@IONPs). OCN/Runx2 was labelled with Cy3 (red), and nuclei were labelled with 4′,6-diamidino-2-phenylindole (DAPI, blue). *p < 0.05, **p < 0.01, ***p < 0.001 compared to the PBS group; ^#^p < 0.05, ^##^p < 0.01 compared to the lipo 2000 group

We conducted a series of assays [alizarin red S (ARS) staining, alkaline phosphatase (ALP) activity examination, and osteogenesis-related gene expression measurements] to assess the osteogenesis ability of the bone mesenchymal stem cells (BMSCs), in which the magnetofection of miR-21 was performed previously. The ARS staining results showed that all four magnetofection groups (lipo 2000, EMF, PEI@IONPs, and EMF-PEI@IONPs groups) exhibited more obvious calcium accumulation than the PBS group (Fig. 3A). Then, the semi-quantification analysis further indicated that there was more mineral deposition in the EMF-PEI@IONPs group than in other groups (Fig. 3B). The analysis of ALP activity showed that the ALP levels in all four magnetofection groups were significantly higher than those in the PBS group and that the EMF-PEI@IONPs group could promote ALP activity better than the lipo 2000 group (Fig. 3C). Furthermore, we performed western blotting (WB) and qPCR experiments to quantify the expression levels of osteogenesis-related genes (Col1, OCN, and OPN). The WB and qPCR results illustrated that in all the four magnetofection groups (lipo 2000, EMF, PEI@IONPs, and EMF-PEI@IONPs groups), the mRNA and protein levels of osteogenesis-related markers (Col1, OCN, and OPN) increased, with the largest increases always being in the EMF-PEI@IONPs group (Fig. 3D–J). Finally, immunofluorescence was employed to detect the expression level of osteogenic markers (OCN and Runx2). All four magnetofection groups exhibited marked enhancement of the OCN and Runx2 expression levels compared to those in the PBS group, with the largest increases being in the EMF-PEI@IONPs group, which were consistent with the WB and qPCR results (Fig. 3K, L). Overall, all four magnetofection methods of miR-21 into BMSCs could enhance their osteogenic differentiation ability, but the greatest effect was observed in EMF-PEI@IONPs group.

### Magnetofection of miR-21 enhances angiogenesis in vitro

**Fig. 4:**
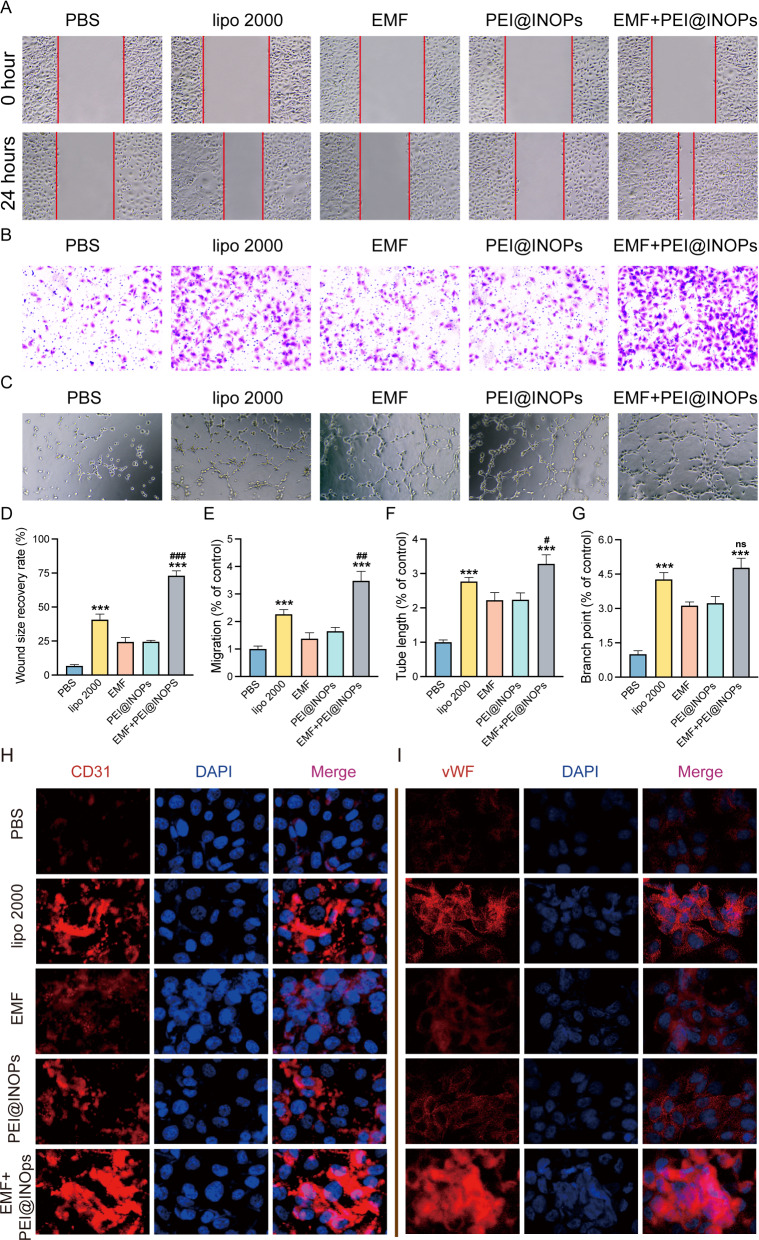
Magnetofection of miR-21 promoted angiogenesis.** A** Assessment of the migratory activity of human umbilical endothelial cells (HUVECs) at 24 h by scratch wound assay; the red dashed lines are the edges of the cell migration. **B** Assessment of the cell migration rate of HUVECs by transwell assay. **C** Tube formation assay of HUVECs. **D**–**G** Quantitative analysis of wound size recovery rate (**D**), migration rate (**E**), total tube length (**F**) and branch points (**G**). *p < 0.05, **p < 0.01, ***p < 0.001 was compared to the PBS group; ^#^p < 0.05, ^##^p < 0.01 was compared to the lipo 2000 group. **H**,** I** Immunofluorescence images of CD31 (**H**) and vWF (**I**) after various treatments. CD31 and vWF was labelled with Cy3 (red), and nucleus were labelled with DAPI (blue)

Angiogenesis and osteogenesis are tightly coupled in bone regeneration. In order to detect the angiogenesis capacity of human umbilical endothelial cells (HUVECs), in which the magnetofection of miR-21 was performed previously, wound-healing experiments, transwell invasion experiments, and tube formation assays were conducted. The images of the wound-healing assay illustrated that the wound area in the four magnetofection groups (lipo 2000, EMF, PEI@IONPs, and EMF-PEI@IONPs groups) was significantly smaller than that of the PBS group after 24 h (Fig. 4A). Further semi-quantification analysis showed that the EMF-PEI@IONPs group exhibited the best wound size recovery rate (Fig. 4D). The results of transwell invasion experiments showed that the EMF-PEI@IONPs group had a faster migration rate than others (Fig. 4B and E). Moreover, results from the tube formation assays showed that there was more tube formation in the lipo 2000 and EMF-PEI@IONPs groups than in the other groups (Fig. 4C). Further semi-quantification analysis indicated that the total tube length in the EMF-PEI@IONPs group was greater than that in the lipo 2000 group. Nevertheless, there were no significant differences in branch points between the lipo 2000 and EMF-PEI@IONPs groups (Fig. 4F, G). Similarly, immunofluorescence results showed that the expression levels of CD31 and vWF increased after treatment with all four magnetofection groups (lipo 2000, EMF, PEI@IONPs, and EMF-PEI@IONPs groups). Specifically, the lipo 2000 and EMF-PEI@IONPs groups increased protein expression levels more than the rest (Fig. 4H, I). In summary, all four magnetofection of miR-21 into HUVECs could promote their angiogenesis capacity, among which the EMF-PEI@IONPs group performed the best.

### Characterization of PCL/HA scaffolds before and after cell seeding

**Fig. 5:**
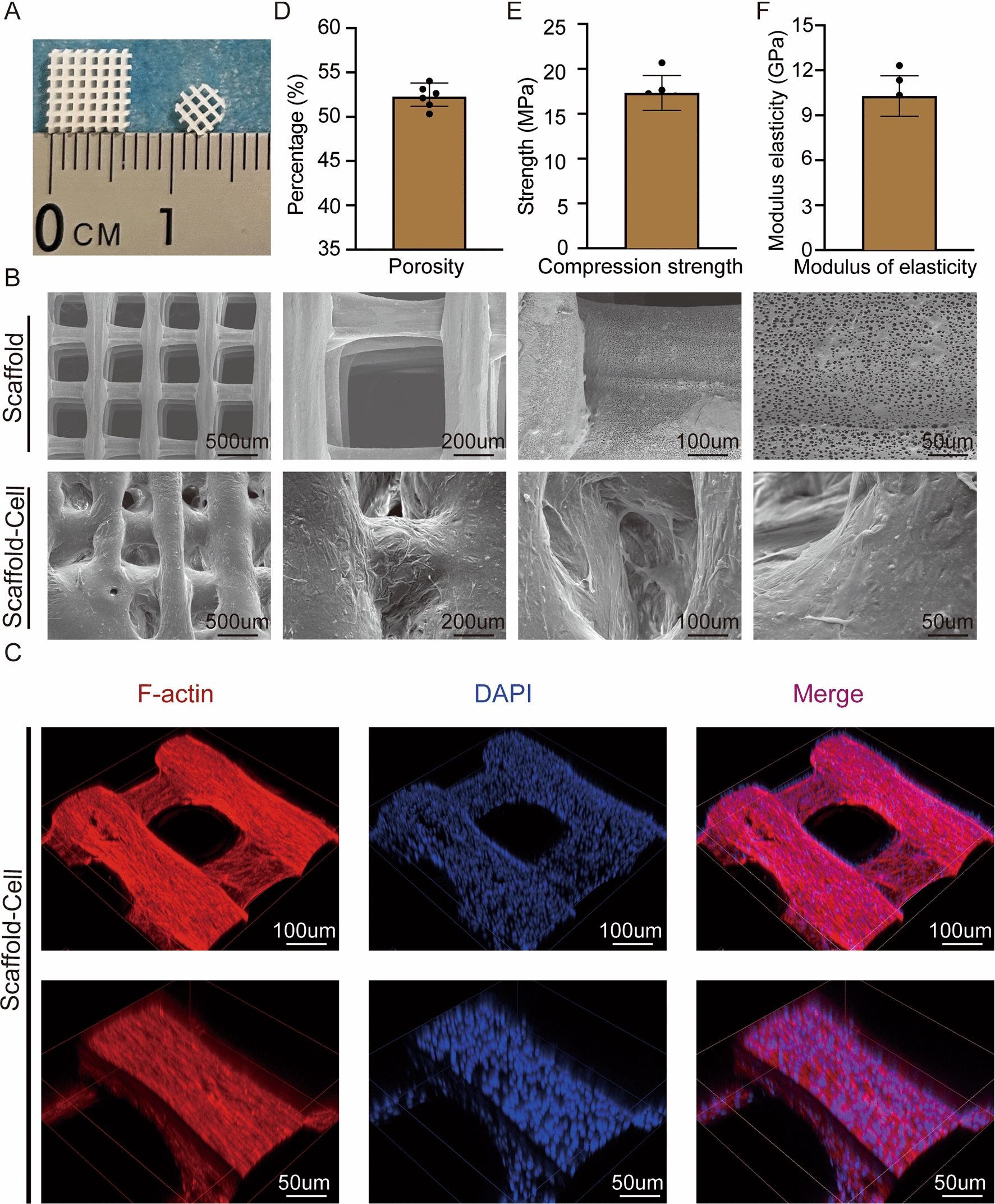
Characterization of polycaprolactone/hydroxyapatite (PCL/HA) scaffolds before and after cell seeding.** A** Gross morphology of PCL/HA scaffolds. **B** Scanning electron microscope (SEM) images showing the macroporous structure of PCL/HA scaffolds at different magnifications (500, 200, 100, 50 μm). The top row shows scaffolds before cell seeding, while the bottom one shows them after cell seeding. **C** Confocal microscope pictures showing cell morphology and distribution. F-action was marked in red and nuclei were marked in blue. **D**–**F** Porosity (**D**), compression strength (**E**), and modulus of elasticity (**F**) of the PCL/HA scaffolds

PCL/HA scaffolds were manufactured with a 3D printer as porous scaffolds with a side length of 5 mm and thickness of 1 mm. Disc-shaped scaffolds with 4 mm in diameter and 1 mm in thickness were used in animal experiments (Fig. 5A). Before cell seeding, the PCL/HA scaffolds uniformly showed a macroporous structure at different scanning electron microscope (SEM) magnifications. The SEM images illustrated that cells were distributed homogeneously on the surface of the PCL/HA scaffolds after cell seeding (Fig. 5B). Confocal microscopy was employed to observe cell morphology and distribution. The results showed that cells with intact F-action cytoskeleton were distributed homogenously along the scaffolds (Fig. 5C). In addition, the 3D-printed PLA/HA composite scaffolds showed an interconnected network of macropores with a porosity of 52.31 ± 2.18% (Fig. 5D). Stress-strain curves suggested that the fabricated scaffolds had a compression strength of 17.33 ± 1.27 MPa (Fig. 5E) and a modulus elasticity of 10.03 ± 1.42 GPa (Fig. 5F). In conclusion, the PCL/HA scaffolds with the macroporous structure were biocompatible and suitable for adherence and proliferation of cells.

### Magnetofection of miR-21-promotes intervertebral fusion in vivo

**Fig. 6:**
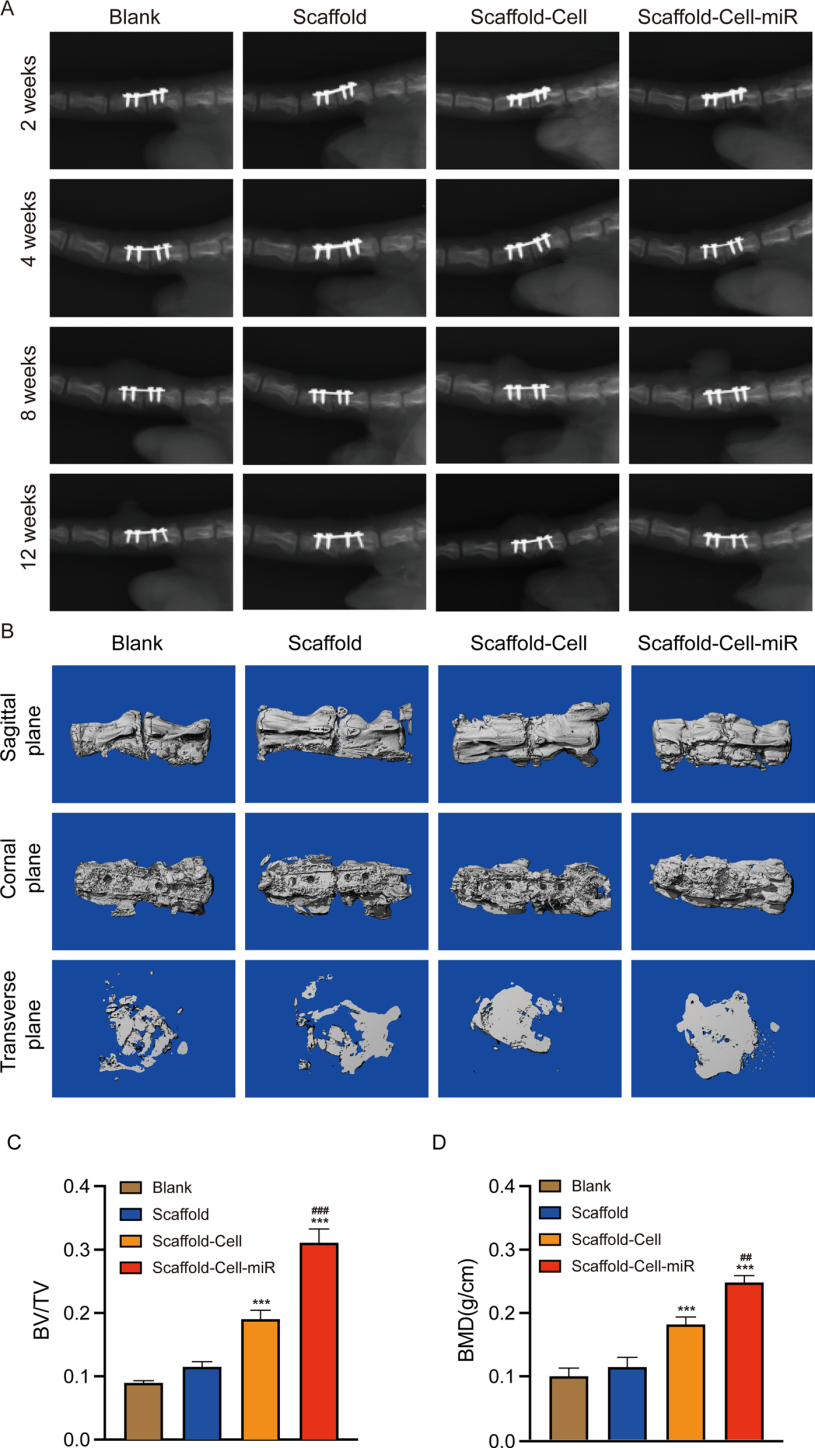
Radiographic assessment (X-ray and micro-CT) of bone regeneration.** A** X-ray images showing the progression of intervertebral fusion 2, 4, 8, and 12 weeks after surgery for the blank, scaffold, scaffold-cell, and scaffold-cell-miR groups. **B** Three-dimensional images reconstructed by micro-CT showing intervertebral fusion conditions under different treatments (blank, scaffold, scaffold-cell, and scaffold-cell-miR groups). The top row shows the sagittal plane; middle, coronal; and bottom, transverse. **C**,** D** Quantification analysis of bone volume relative to total volume (BV/TV) (**C**), and bone mineral density (BMD) (**D**). *p < 0.05, **p < 0.01, ***p < 0.001 compared to the blank group. ^#^p < 0.05, ^##^p < 0.01 compared to the scaffold-cell group

X-rays were employed to monitor the progression of intervertebral fusion from 2 to 12 weeks. The X-ray images showed that all four groups (blank, scaffold, scaffold-cell, and scaffold-cell-miR groups) exhibited fusion as time passed from 2 to 12 weeks. The scaffold-cell and scaffold-cell-miR groups promoted intervertebral fusion better than the other two groups (blank and scaffold groups) (Fig. 6A). Micro-CT was employed to identify the fusion conditions and construct three-dimensional images of two adjacent vertebrae. The results demonstrated that the intervertebral fusion conditions progressed significantly better after treatment with the scaffold-cell and scaffold-cell-miR groups compared with those in the other two groups (blank and scaffold groups) at 12 weeks, with the best fusion conditions being observed in the scaffold-cell-miR group (Fig. 6B). Further quantification analysis of bone volume relative to total volume (BV/TV) and bone mineral density (BMD) showed that there was more newly-formed bone in the scaffold-cell and scaffold-cell-miR groups compared with that in the other two groups (blank and scaffold groups), with the scaffold-cell-miR group having the highest amount of newly-formed bone (Fig. 6C, D).

**Fig. 7:**
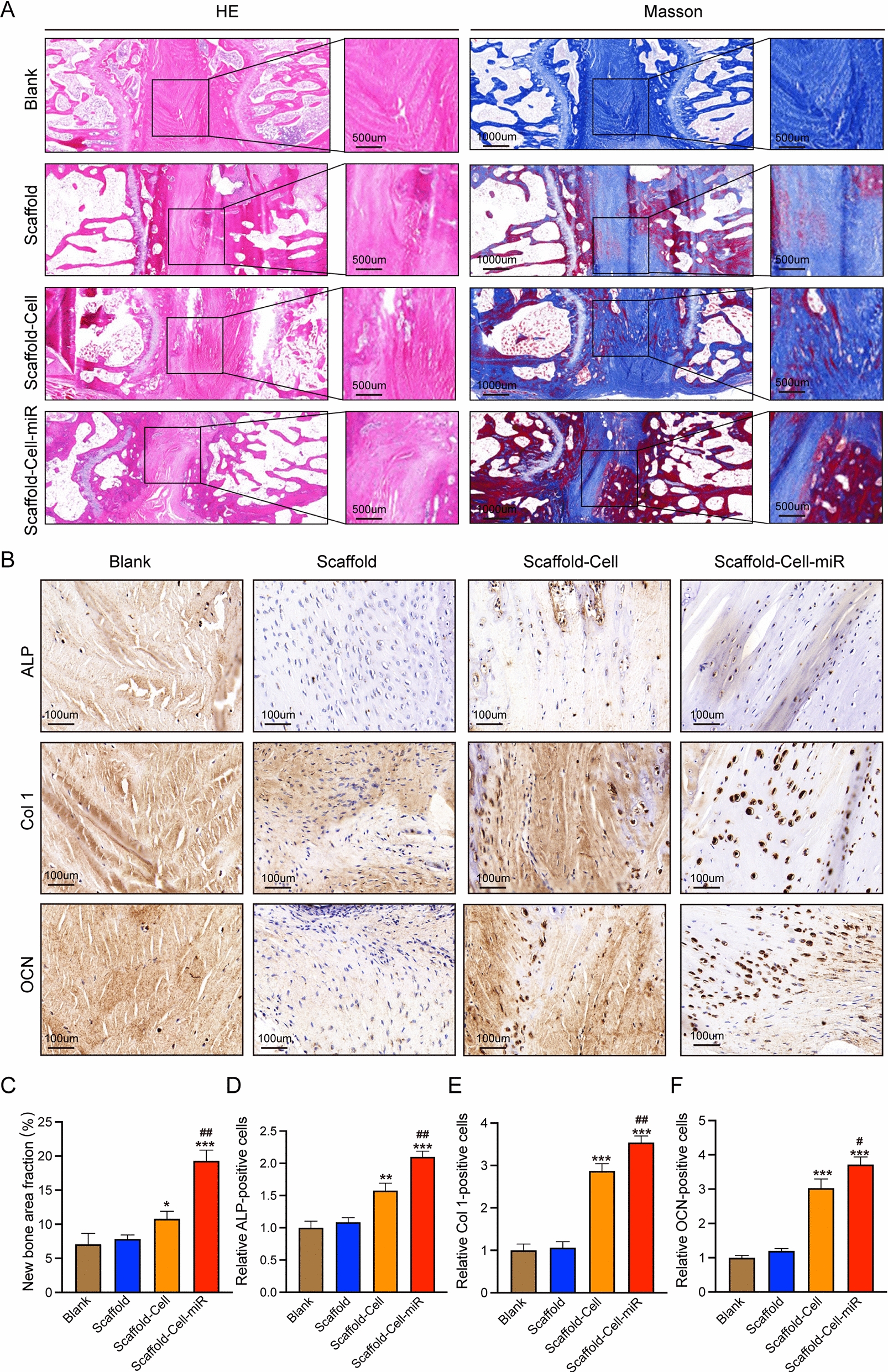
Histological verifications [haematoxylin and eosin (HE), Masson, and immunohistochemical staining analysis] of bone regeneration.** A**,** C** HE and Masson staining images detecting the intervertebral fusion condition (**A**), and corresponding new bone area fraction analysis (**C**). **B**,** D–F** Immunohistochemical staining of the osteogenic markers alkaline phosphatase (ALP), Col 1, and OCN (**B**) and corresponding quantification of ALP (+) (**D**), Col 1(+) (**E**), and OCN (+) (**F**) cells

Following the radiographic experiments (X-ray and micro-CT reconstruction), a series of histological verifications were conducted. The results from haematoxylin and eosin (HE) and Masson staining showed that all four groups exhibited collagen formation. Significantly, we found that there was more new mature bone formation (stained in red in Masson staining) in the scaffold-cell and scaffold-cell-miR groups (Fig. 7A). Further semi-quantification results verified the above points on new bone area formation (Fig. 7C). The images and semi-quantification results from immunohistochemical staining for ALP, Col 1, and OCN showed that there were more ALP (+), Col 1 (+), and OCN (+) cells observed in the scaffold-cell and scaffold-cell-miR groups (Fig. 7B and D–F). To summarise, all aforementioned radiographic and histological evidence indicated that the magnetofection of miR-21 enhanced the amount of newly-formed bone and promoted intervertebral fusion in vivo.

### EMF enhances magnetofection efficiency through the p38 MAPK pathway

**Fig. 8:**
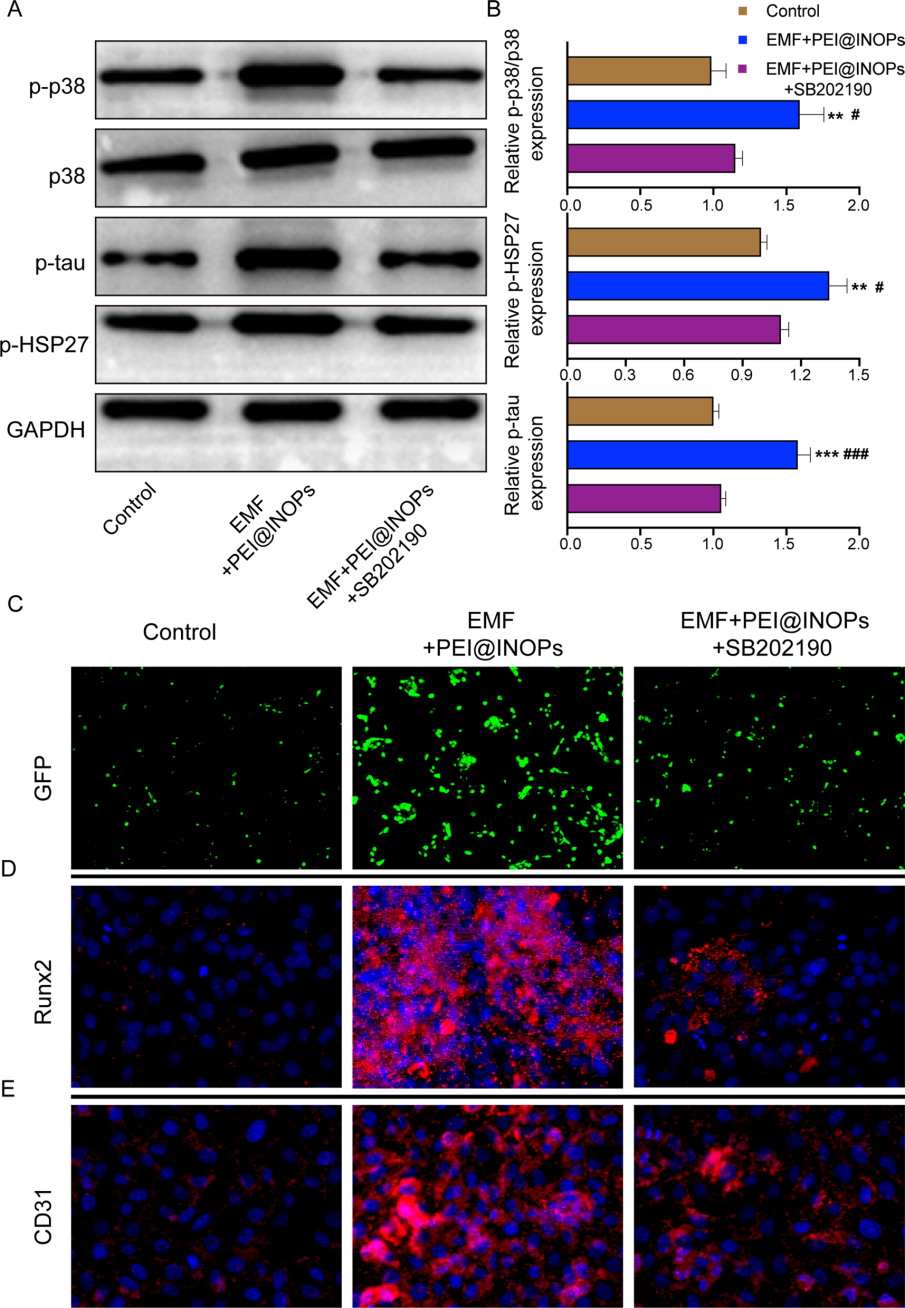
Electromagnetic field (EMF) enhanced transfection efficiency through the activation of p38 MAPK pathway.** A**,** B** Western blotting bands and corresponding protein expression levels of phosphorylated p38, p38, tau, and HAP27 (p-p38, p38, p-tau, p-HSP27, respectively). *p < 0.05, **p < 0.01, ***p < 0.001 compared to the control group. ^#^p < 0.05, ^##^p < 0.01 compared to the EMF + PEI@IONPs + SB202190 group. **C** Fluorescence microscope images showing different transfection efficiencies under different treatments (control, EMF + PEI@IONPs, and EMF + PEI@IONPs + SB202190). **D**,** E** Immunofluorescence images of Runx2 (D) and CD31 (E) after various treatments (control, EMF + PEI@IONPs, and EMF + PEI@IONPs + SB202190). Runx2/CD31 was labelled with Cy3 (red), and nucleus were labelled with 4′,6-diamidino-2-phenylindole (DAPI, blue)

Our previous studies showed that EMF was associated with the activation of the p38 MAPK pathway [48, 49]. Therefore, we hypothesised that EMF promoted magnetofection of miR-21 into seed cells via p38 MAPK pathway. To verify this hypothesis, we employed SB202190 as an inhibitor of the p38 MAPK pathway and conducted a series of rescue experiments. Western blotting results showed that EMF significantly increased protein expression levels of phosphorylated p38, tau, and HSP27 (p-p38, p-tau, and p-HSP27, respectively), indicating the activation of the p38 MAPK pathway by EMF. In addition, SB202190 could attenuate the p38 MAPK pathway activation stimulated by EMF and significantly decrease the protein expression levels of p-p38, p-tau, and p-HSP27 (Fig. 8A, B). The results from immunofluorescence imaging confirmed that the positive effect on transfection efficiency, osteogenesis, and angiogenesis of EMF could be attenuated by SB202190, an inhibitor of the p38 MAPK pathway (Fig. 8C–E). All the above results indicated that EMF enhanced magnetofection efficiency through the p38 MAPK pathway and promoted osteogenesis and angiogenesis.

## Discussion

Silencing specific disease-causing genes via the miRNAs sponge effect has shown great potential for clinical applications. Recent studies have characterised miR-21 to be closely related to many biological processes, especially to osteogenesis and angiogenesis [50–53]. For example, Geng et al. reported that miR-21 could promote bone formation by enhancing angiogenic and osteogenic differentiation in mesenchymal stem cells [54]. Here, we found that miR-21 transfected into BMSCs and HUVECs could simultaneously promote osteogenesis and angiogenesis. Therefore, miR-21 delivery could be a potential strategy to promote the formation of bone and blood vessels.

Despite their therapeutic potential for bone formation, successful and efficient delivery of miRNAs still poses a tremendous challenge. Viral vectors are excellent carriers for delivering miRNAs, but they also have disadvantages, such as immunogenicity and manufacturing costs, that limit their further application [16, 17, 55, 56]. Consequently, magnetofection methods for gene delivery have attracted considerable attention due to their safety and high efficiency [23, 25]. In this study, magnetofection was employed to deliver miR-21, and the transfection efficiency results proved to be encouraging.

There are various magnetic systems employed in magnetofection for gene delivery. Among these, static magnetic fields generated by permanent magnets are the most widely used to attract IONP adherence to cells [25, 57–59]. In this study, we substituted EMF for a permanent magnet with a physical adsorption effect. EMF is a widely used, non-invasive method to promote osteogenesis and angiogenesis [60]. Here, our results indicated that EMF enhanced magnetofection efficiency significantly and that it was mediated through the activation of the p38 MAPK signalling pathway. Therefore, EMF is a good substitute for magnets and promotes transfection efficiency via the p38 MAPK pathway.

Finally, we show that the magnetofection of miR-21 into BMSCs and HUVECs could guide cytologic behaviour and promote osteogenic and angiogenic differentiation in seed cells and that, once planted on PCL/HA scaffolds, these engineered cells could progress into idealized engineering bone. Moreover, the combination of EMF and IONPs enhanced magnetofection efficiency significantly via the activation of the p38 MAPK signalling pathway. Nevertheless, we should state that there were some limitations to this study. Firstly, the specific molecular mechanism by which EMF promotes transfection efficiency remains unclear. Secondly, a more variety of cells is needed to examine the stability of our magnetofection system. Thus, further studies are needed.

## Conclusion

Our study described a novel, nontoxic, biocompatible, and robust magnetofection system for delivering therapeutic miRNAs to target seed cells. We had previously identified a miRNA, miR-21, crucial for osteogenesis and angiogenesis. Then, the magnetofection systems (EMF- and PEI@IONPs-bound miRNAs) were employed to modulate the behaviour of target seed cells. PCL/HA scaffolds were then applied as a vehicle for these engineered seed cells. Ultimately, we constructed an ideal tissue-engineered bone to achieve interbody fusion successfully in a rat intervertebral fusion model. Finally, further results indicated that EMF and IONPs enhanced the transfection efficiency via the activation of the p38 MAPK pathway. In conclusion, this technology could contribute to the development the miRNA-based gene therapy to treat various orthopaedic diseases.

## Methods

### Preparation of magnetofection gene complexes

Firstly, IONPs were synthesized via the co-precipitation of ferrous and ferric ions in an alkaline medium. Afterwards, we mixed the PEI and IONPs together at a mass ratio of 5:1 to coat PEI onto the surface of IONPs. For the preparation of magnetofection gene complexes, plasmid-miRNA and PEI@IONPs were mixed at various ratios of PEI@IONPs:miRNA (i.e., 5, 10, 20, and 30) and incubated for 30 min to form magnetofection gene complexes.

The hydrodynamic size and zeta potential analyses of PEI@IONPs were performed using a Zetasizer Nano ZS instrument (Malvern, Worcestershire, UK). The reproducibility of diameter and zeta potential was confirmed by measuring three independent samples. The morphologies and intracellular distribution of were detected by transmission electron microscopy (TEM, Jeol TEM-2010; Tokyo, Japan).

### Electromagnetic field

The equipment for generating sinusoidal electromagnetic fields was fabricated by the Naval University of Engineering (Wuhan, China). The EMF equipment was composed of a waveform generator for generating a sinusoidal electromagnetic field signal, an amplifier for amplifying the sent signal, and a pair of Helmholtz coils. The frequency and strength of the generated electromagnetic field were confirmed by an oscilloscope.

### Cell culture and cell proliferation assays

Sprague-Dawley rat BMSCs, HUVECs, and 293T cells were purchased from Cyagen Biosciences Inc. (Suzhou, China). BMSCs and 293T cells were cultured in DMEM/F12 medium (Gibco, NY, USA) containing 10% foetal bovine serum (FBS, Gibco, NY, USA) and 1% of an antibiotic-antimycotic solution (Sigma-Aldrich, NY, USA). HUVECs were cultured in endothelial basal medium (EBM-2, Lonza, Switzerland) containing endothelial growth supplement (EGM-2). All the cells were maintained at 37 °C in a humidified incubator with 5% CO_2_.

Cell proliferation was assessed with a Cell Counting Kit-8 (CCK-8, Boster, China). The proliferation of seed cells was measured after 1, 3, and 5 days following the manufacturer’s instructions.

### Alizarin red staining and ALP activity

Alizarin red staining (ARS) and ALP activity were conducted to detect the formation of mineralized matrices. ARS was conducted for differentiated BMSCs following the manufacturers’ instructions. BMSCs were fixed using 95% (v/v) ethanol for 10–15 min and then stained with 2% (v/v) ARS solution for 30 min at 37 °C. Then, they were washed thrice with distilled water to remove any excess dye. ALP activity, highly correlated with bone formation, was determined using an alkaline phosphatase assay kit. Firstly, an alkaline buffer solution was added into cell lysates and then incubated for 5–10 min at 37 °C. Finally, ALP activity was normalised with the standard curve.

### Scratch-wound and transwell assays

Scratch-wound and transwell assays were performed to determine the cell migration capacity of HUVECs. Firstly, HUVECs were cultured in EBM-2 supplemented with EGM-2. Then, the scratch-wound assay was performed from top to bottom of the cell monolayer, and the cells in this area were removed by washing them with PBS. Wound size was measured at 0 and 24 h after the start of the assay. The wound size recovery rate (%) was calculated through the formula: (wound size at 0 h – wound size at 24 h)/wound size at 0 h ×100. As for the transwell assay, HUVECs were planted in the upper chambers and incubated for 24 h. The migration rate (% of control) of the transwell assay was obtained with the migration rate of the PBS group as a baseline.

### Tube formation assay

Tube formation assays were conducted to detect the angiogenic capability of HUVECs under various conditions in vitro. Briefly, HUVECs were seeded in the gel and incubated at 37 °C and 5% CO_2_ for 4 h. Tube length and branch points were calculated using ImageJ software to assess the tube formation.

### qRT‑PCR analysis

RNA was extracted using TRIzol, and the RNA concentration was determined by a NanoDrop-2000 spectrophotometer (Thermo Fisher Scientific, MA, USA). The extracted RNA was reverse-transcribed into cDNA with the stem-loop reverse transcriptase primer Kit (Ribobio, Guangzhou, China). Gene expression was measured using a Bio-Rad myiQ2 thermal cycler (Bio-Rad, Hercules, CA, USA) with the SYBR Prime Script kit (Takara Bio Inc., Shiga, Japan). All experiments were repeated three times.

### Western blotting

Cells were lysed, and the concentrations of their extracted proteins were determined by the BCA Protein Assay kit (Beyotime, Shanghai, China). The proteins were separated by sodium dodecyl sulphate-polyacrylamide gel electrophoresis (SDS-PAGE) and then transferred to polyvinylidene difluoride (PVDF) membranes. Thereafter, the membranes were treated with 5% (v/v) skimmed milk in TRIS-buffered saline with 0.1% (v/v) Tween-20 (TBST) for 60 min. The sealed membranes were incubated with specific antibodies at 4 °C overnight. The primary antibodies against OCN, ALP, OPN, Runx2, COL-1, vWF, VEGF, and CD31 were purchased from Cell Signaling Technology (Beverly, MA, USA) and Abcam (Cambridge, UK).

### Rat intervertebral fusion model

All animal procedures were approved by the Animal Care and Use Committee of Huazhong University of Science and Technology. Firstly, a total of 2 × 10^5^ cells (BMSCs: HUVECs = 1:1) were cultured in a 24-well plate with PCL/HA scaffolds with or without EMF-assisted magnetofection of miR-21 to prepare cell-scaffold complexes. Rats were anaesthetized by intraperitoneal injection of 1% (w/v) pentobarbital sodium (40 mg/kg), and the surgical area was disinfected with iodophor. Then, the skin and subcutaneous tissue were incised gently to expose the vertebral bodies. Afterwards, a scalpel was used to remove intervertebral discs and soft tissue completely. Then, rats in different groups were administered their respective treatments as indicated. Finally, after the two adjacent vertebrae were fixed, the incision was closed. The surgical area was disinfected with iodophor, followed by antibiotic application for prophylaxis.

According to the difference of treatments, twenty-four Sprague Dawley rats (10 weeks old, male, 300–350 g) were randomly assigned into four groups: (1) blank group: the interbody space was implanted with nothing; (2) scaffold group: the interbody space was implanted with only scaffolds; (3) scaffold-cell group: the interbody space was implanted with the cell-scaffold complexes without EMF-assisted magnetofection of miR-21; and (4) scaffold-cell-miR group: the interbody space was implanted with EMF-assisted magnetofection of miR-21.

### Fabrication and characterisation of PCL/HA scaffolds

PCL and HA were dissolved and mixed at a ratio of 7:3, and dichloromethane (Sigma-Aldrich) was used as a solvent. Subsequently, the mixture was printed into porous scaffolds using a fused deposition modelling 3D printer. The fabricated 3D-printed PCL/HA scaffolds were sterilized with ethylene oxide gas before use.

The morphology of the PCL/HA scaffolds was observed with a scanning electron microscope (SEM, SU8100, HITACHI). Briefly, the fabricated scaffolds were first fixed and then dehydrated through an alcohol series. Finally, after drying and conductive metal coating, they were observed and taken images with the SEM.

A drainage method was performed to assess the porosity of the PCL/HA scaffolds, while mechanical properties were determined with an Instron 5566 (Instron corporation, USA).

### X-ray and Micro-CT analysis

Intervertebral fusion conditions were evaluated by X-ray (voltage 50 kV, current 160 mA, exposure time 63 ms). The morphology of vertebral bodies was scanned with Micro-CT (vivaCT 40, Scanco 274 Medical, Switzerland). The reconstruction of the 3D images and determination of the bone volume relative to total volume (BV/TV) and the bone mineral density (BMD) were conducted with Mimics software (Materialise, Leuven, Belgium).

### Statistical analysis

All experiments were performed a minimum of three times. Student’s t-test was employed to test comparisons between two groups, and one-way analysis of variance (ANOVA) was conducted to compare differences among multiple groups. Statistical significance is stated as (*, #) p < 0.05, (**, ##) p < 0.01 and (***, ###) p < 0.001.

## Data Availability

The datasets used and/or analyzed during the current study are available from the corresponding author on reasonable request.

## Abbreviations

ALP: Alkaline phosphatase
ARS staining: Alizarin red S staining
BMD: Bone marrow density
BMSCs: Bone marrow mesenchymal stem cells
BTE: Bone tissue engineering
BV/TV: Bone volume relative to total volume
DAPI: 4′,6-Diamidino-2-phenylindole
DDD: Degenerative disc disease
EMF: Electromagnetic field
HA: Hydroxyapatite
HE staining: Haematoxylin and eosin staining
HUVECs: Human umbilical endothelial cells
IONPs: Iron oxide nanoparticles
lipo 2000: Lipofectamine 2000
MFI: Mean fluorescence intensity
PBS: Phosphate-buffered saline.
PCL: Polycaprolactone
PEI: Polyethyleneimine
SEM: Scanning Electron Microscope
WB: Western blotting
